# Supplementary material for: Programmed Cell Death-1/Programmed Cell Death-1 Ligand as Prognostic Markers of Coronavirus Disease 2019 Severity
Source: Cells. 2022 Jun 20;11(12):1978. doi: 10.3390/cells11121978 (PMC9222173; doi:10.3390/cells11121978)
Supplement: Supplementary file 1 [file cells-11-01978-s001.zip › cells-1737772-supplementary.pdf]

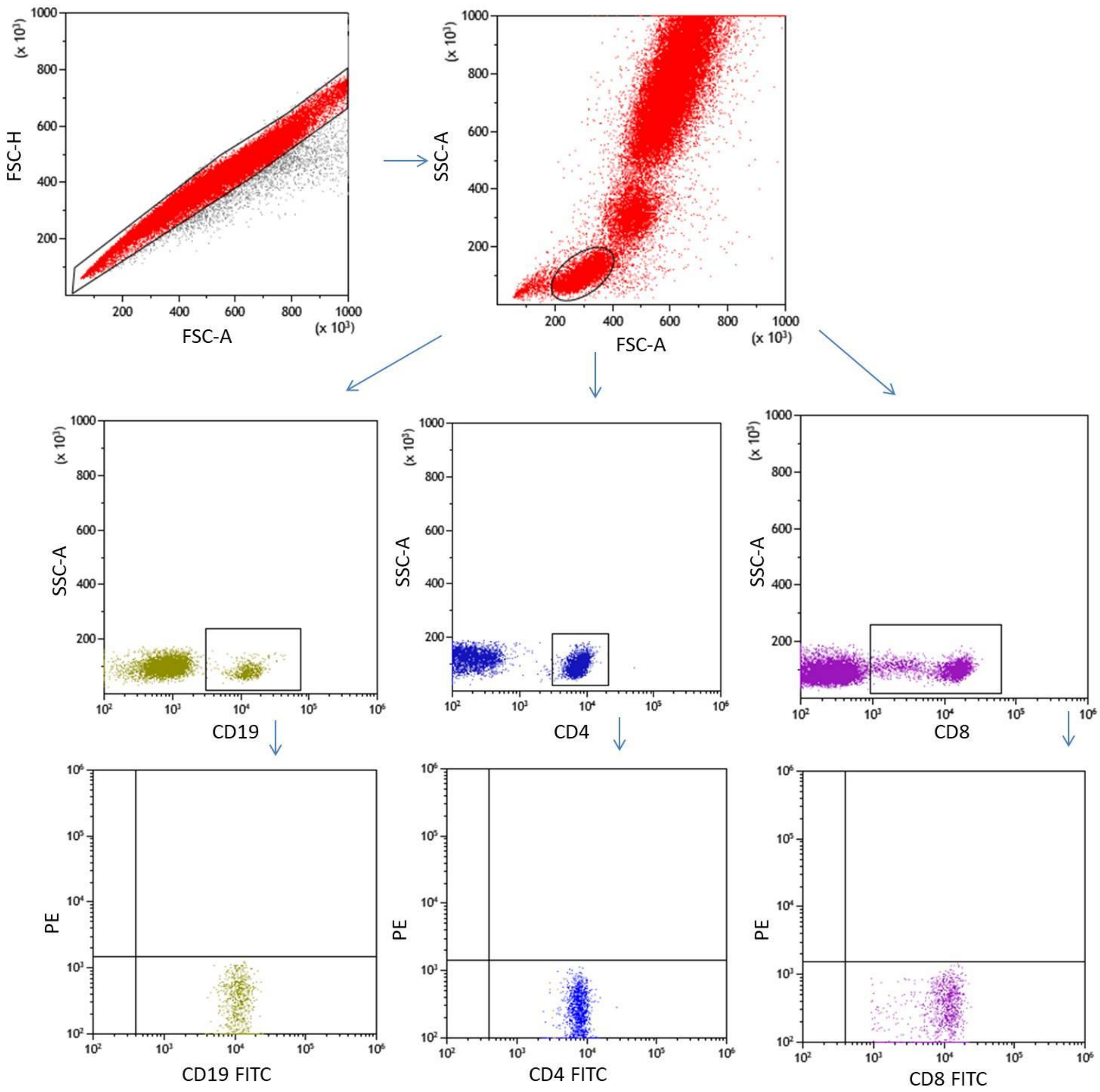

Figure S1. FMO control for cells stained with FITC and PE conjugated antibodies (PE - no stain).

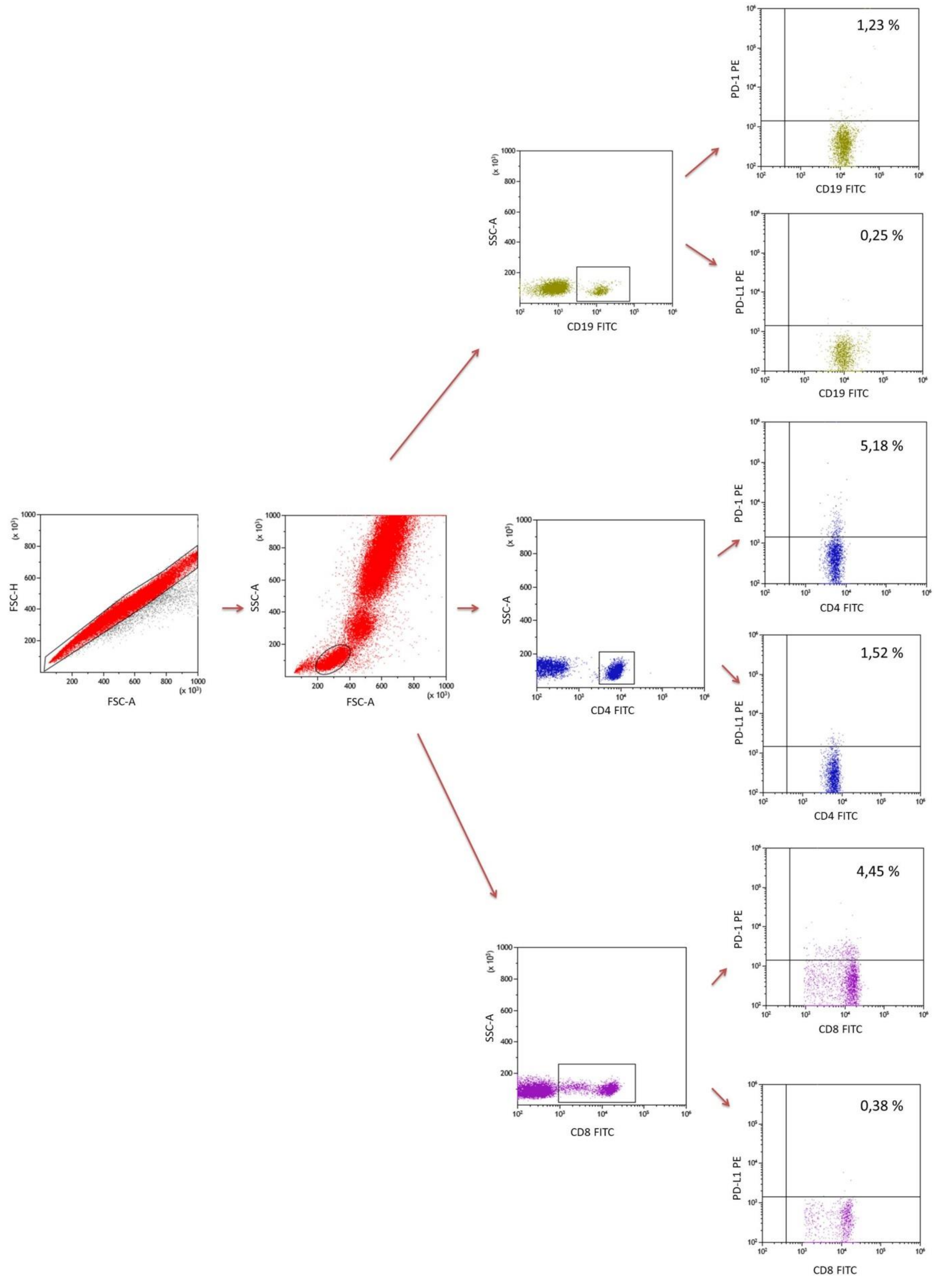

Figure S2. Cytometric analysis of PD-1-positive and PD-L1- positive lymphocytes in healthy controls.

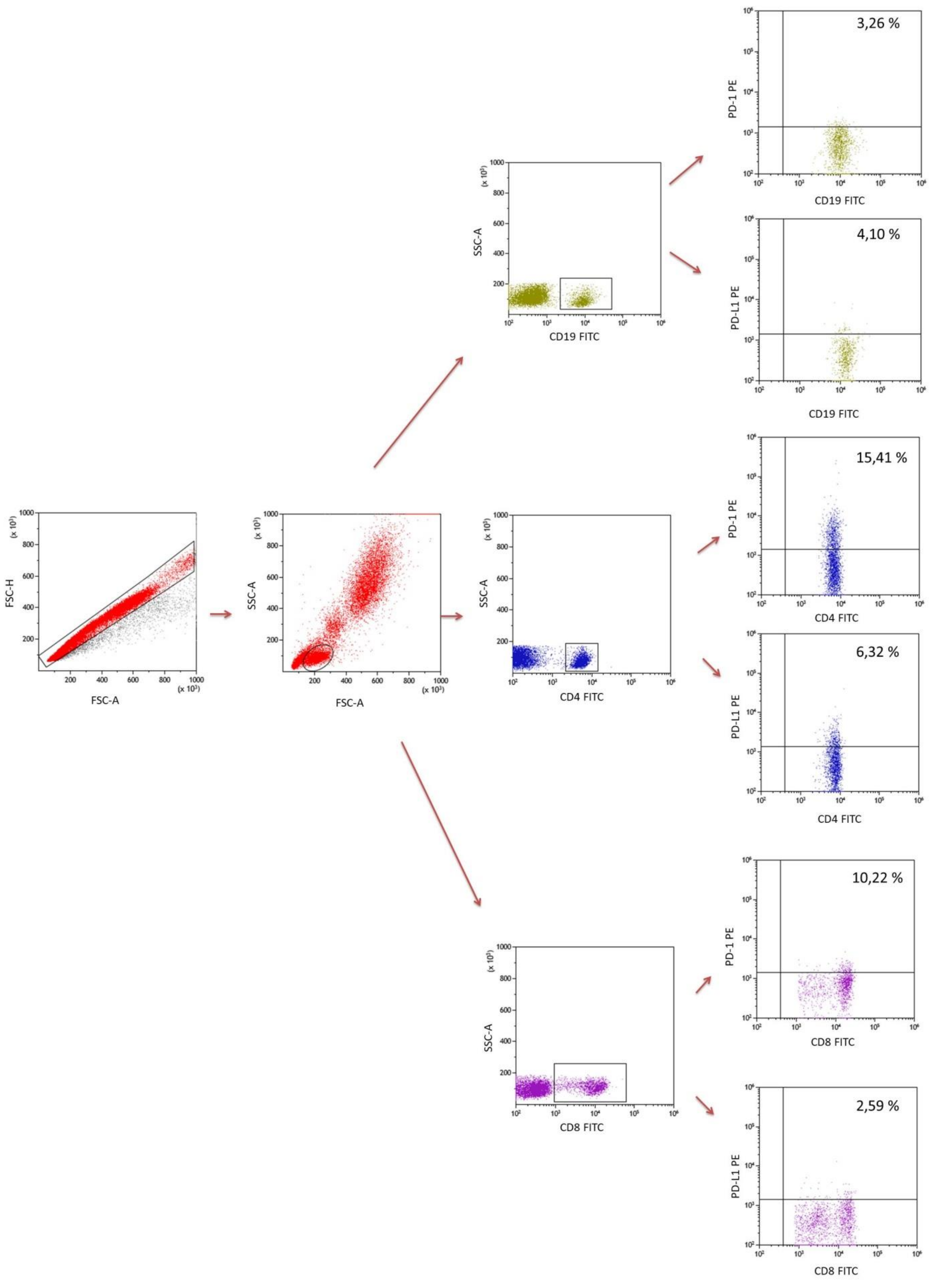

Figure S3. Cytometric analysis of PD-1-positive and PD-L1- positive lymphocytes in patients with COVID-19 hospitalized in the non-ICU

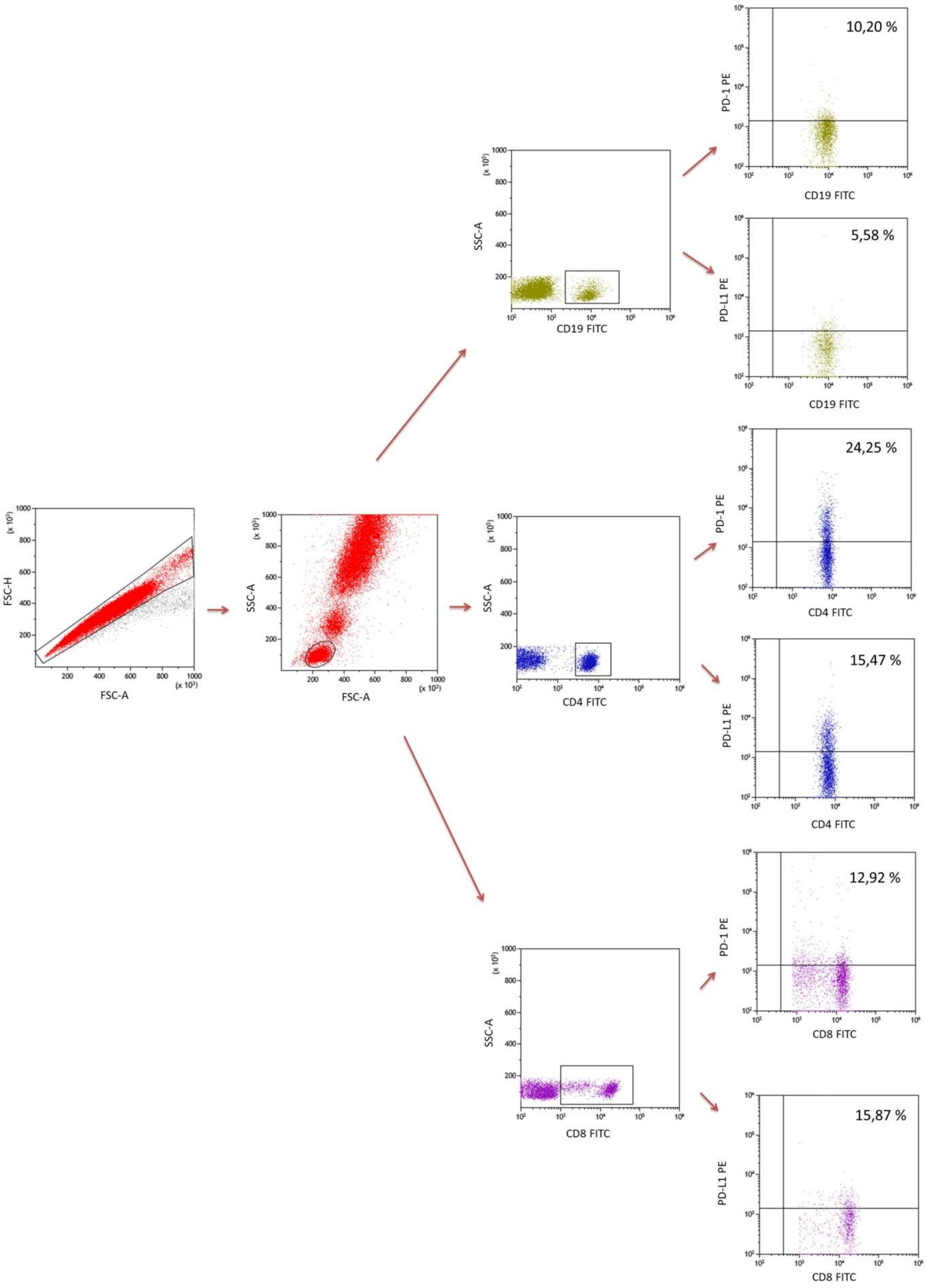
FSC-H
FSC-A
SSC-A
FSC-A
SSC-A
CD19 FITC
PD-1 PE
CD19 FITC
10,20 %
PD-L1 PE
CD19 FITC
5,58 %
SSC-A
CD4 FITC
PD-1 PE
CD4 FITC
24,25 %
PD-L1 PE
CD4 FITC
15,47 %
SSC-A
CD8 FITC
PD-1 PE
CD8 FITC
12,92 %
PD-L1 PE
CD8 FITC
15,87 %

Figure S4. Cytometric analysis of PD-1-positive and PD-L1- positive lymphocytes in patients with COVID-19 hospitalized in the ICU

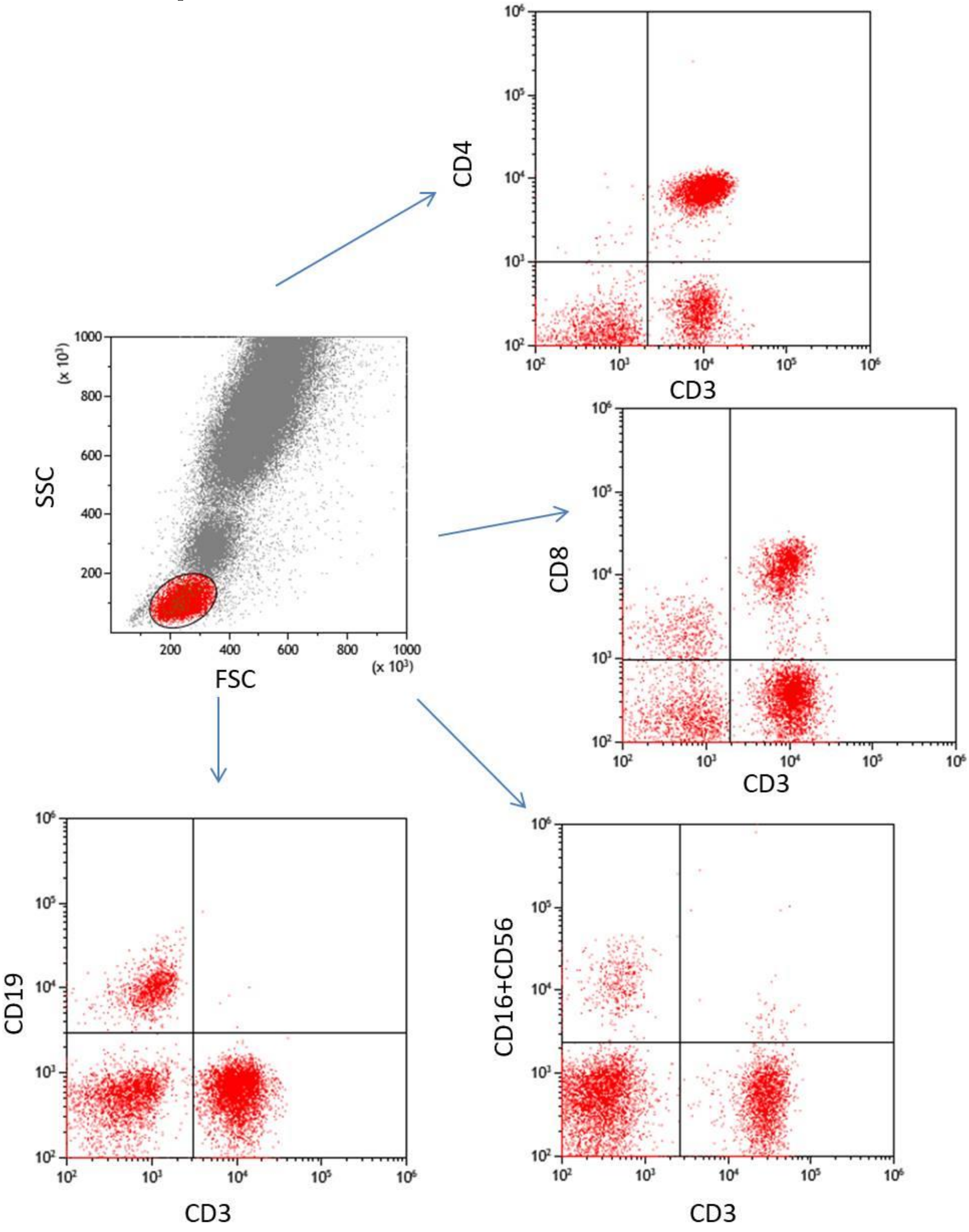

Figure S5. Sample analysis of lymphocytes.
